# Testing and Modelling of Elastomeric Element for an Embedded Rail System

**DOI:** 10.3390/ma14226968

**Published:** 2021-11-18

**Authors:** Qianqian Li, Roberto Corradi, Egidio Di Gialleonardo, Stefano Bionda, Andrea Collina

**Affiliations:** Department of Mechanical Engineering, Politecnico di Milano, Via La Masa 1, 20156 Milan, Italy; roberto.corradi@polimi.it (R.C.); egidio.digialleonardo@polimi.it (E.D.G.); stefano.bionda@polimi.it (S.B.); andrea.collina@polimi.it (A.C.)

**Keywords:** elastomeric material, preload-dependent damping, frequency-dependent damping, macro-mechanical modelling of damping, embedded rail system

## Abstract

Modelling of elastomeric elements of railway components, able to represent stiffness and damping characteristics in a wide frequency range, is fundamental for simulating the train–track dynamic interaction, covering issues such as rail deflection as well as transmitted forces and higher frequency phenomena such as short pitch corrugation. In this paper, a modified non-linear Zener model is adopted to represent the dependences of stiffness and damping of the rail fastening, made of elastomeric material, of a reference Embedded Rail System (ERS) on the static preload and frequency of its deformation. In order to obtain a reliable model, a proper laboratory test set-up is built, considering sensitivity and frequency response issues. The equivalent stiffness and damping of the elastomeric element are experimentally characterised with force-controlled mono-harmonic tests at different frequencies and under various static preloads. The parameters of the non-linear Zener model are identified by the experimental equivalent stiffness and damping. The identified model correctly reproduces the frequency- and preload-dependent dynamic properties of the elastomeric material. The model is verified to be able to predict the dynamic behaviour of the elastomeric element through the comparison between the numerically simulated and the experimentally measured reaction force to a given deformation time history. Time domain simulations with the model of the reference ERS demonstrate that the modelled frequency- and preload-dependent stiffness and damping of the elastomeric material make a clear difference in the transient and steady-state response of the system when distant frequency contributions are involved.

## 1. Introduction

Railway transport is credited to be one of the best solutions for helping in the reduction of environmental air pollution and land use, in comparison with its transport capacity for both passengers and goods. On the other hand, especially when interacting in an urban context, the aerial noise emission and transmission of vibration to residential and commercial buildings nearby is one of the issues related to the environmental impact of railways. The involved frequency range is usually from 10 Hz to 250 Hz for the vibrations transmitted through the ground (whether for a surface line or in a tunnel), originating direct vibrational disturbance and secondary noise. A higher frequency range is involved in direct noise emission (rolling noise).

To study these issues, numerical train–track interaction models have been widely employed. Such models usually include three main sub-models: the vehicle model, the track model, and the contact force model [1,2]. According to the characteristics of the main phenomenon and the type of train or track under study, the specific compositions of each sub-model vary correspondingly as well as the requirements for their ability to correctly represent the dynamic properties of the physical systems or components.

Concerning the track model, it would be ideal to properly simulate, with a unique model, the dynamic behaviour of the track in a relatively wide frequency range, in order to cope, at the same time, both with issues related to rail deflections (low-frequency range), to transmitted vibration and noise generation (medium- to high-frequency range). The concerned frequency ranges of different issues are listed in Table 1.

Rail deflection is confined in the low-frequency range, associated with the ratio between train speed and bogie wheelbase, i.e., in the range 5 ÷ 20 Hz for speed 30 ÷ 200 km/h and wheelbase 1.8 ÷ 3 m. On the contrary, transmitted forces associated with vibrational problems are indicatively in the frequency range 10 ÷ 250 Hz. However, in this large frequency range, not all track components have constant dynamic properties. The elastomeric elements, for example, which are generally used in fastening systems, have frequency-dependent stiffness and damping [3,4,5,6,7,8,9]. The rail fastening system is a crucial part of the track because the forces coming from wheel–rail contact are first distributed by the rail, and then transmitted through the fastening system to the tunnel invert in the case of a direct connection, to a slab in the case of a slab track system, or to sleepers in the case of a classical ballasted track system. While the dependence of stiffness and damping on frequency is relatively easy to model within a frequency domain approach, to reproduce them in time domain requires suitable models, such as Zener or other similar rheological models. In addition, dynamic behaviour of elastomeric elements can be also dependent on the static preload [3,4,7,8,10].

It is also recognised that the dynamic properties of the elastomeric elements could have a dependence on temperature [11,12] as other polymer-based products [13,14,15]. Nevertheless, except the installation in the regions where the environment temperature experiences a large variation during the operation time, it is regarded that the effect of the dependence of the dynamic properties on the temperature is relatively small on the train or track responses since the frequency of the train passages is not going to significantly increase the temperature of the elastomeric elements.

For a classical time domain train–track interaction model, the frequency- and preload-dependent dynamic behaviour of elastomeric elements is not taken into consideration and the linear Kelvin–Voigt model is usually employed [1,2,16,17,18]. Several approaches have been studied by various researchers to model the non-linear dynamic behaviour of the elastomeric elements. Dahlberg [19] described the stiffness of a railpad in function of the second order of its compression. Similarly, Kargarnovin et al. [20] used a cubic function to model the stiffness of a non-linear viscoelastic foundation. Uzzal et al. [21] used an exponential function to describe the stiffness–deformation relationship of rail pads. Andersson et al. [22] used a three-parameter Zener model to reproduce the dependence of the stiffness and damping on frequency while Johansson et al. [7] used a four-parameter viscoelastic model (two Kelvin–Voigt models in series) for the same aim where different sets of parameters values are identified for different static preloads. Berg [23] and Bruni et al. [24] described the dependence of the dynamic properties on frequency and deformation amplitude with rheological models. De Man [25], Maes et al. [9], and Koroma et al. [26] used the modified three-parameter Zener model for the dependence on frequency and static preload. Fenander [27] and Zhao et al. [28] used fractional models for the same objective. Nonetheless, due to the limited research of the concerned topic, it has not been verified whether the proposed models can be applied to different elastomeric elements used in different tracks. For instance, most of the foregoing studies concern the rail pads. Furthermore, the effect of the non-linear dynamic behaviour of the elastomeric element on the train–track dynamic interaction has not been comprehensively studied.

It is useful to develop a model of the non-linear dynamic properties of the elastomeric elements in function of the static preload, and at the same time, the dependence on frequency, for the time domain train–track dynamic interaction. With such a model, it is possible to investigate in a single simulation the aspects of the train running safety, such as the track gauge widening and the rail deflection, which are low-frequency phenomena, and the high-frequency ones related to the transmission of the vibration and railway noise. For such a model, it is important to correctly reproduce not only the nonlinearity of the stiffness of the elastomeric material, which is fundamental for the dynamic responses of trains and tracks, but also the damping, which has a significant influence on the magnitudes of the responses.

The main aim of the paper is to adopt a simple model for the frequency- and preload-dependent stiffness and damping of elastomeric elements based on a modified Zener model, which is to be easily used in the time domain simulation of train–track interaction. It is also desired that the developed model is computationally efficient, so as to facilitate the time domain simulation of train–track interaction. To this end, the rail fastening system of an Embedded Rail System (ERS), which is made of elastomeric material, was chosen as a reference elastomeric element for the development of the model. The ERS is a configuration aiming to better distribute the transmitted forces originating from the wheel–rail contact, which is usually adopted for tramway lines and in some cases proposed for metro lines and metallic bridges. An ERS is composed of an ordinary rail, embodied in a volume of elastomeric material, that can be of different nature and composition. The latter is inserted in a longitudinal groove that can be obtained in a concrete slab or built as a steel channel. An under-rail pad can be optionally adopted. Moreover, empty volumes can also be realised in the elastomeric volume, inserting plastic tubes, in order to save material and to optimise the ratio between the lateral and rotational stiffness. The purpose of this assembly is to provide a continuous vertical, lateral, and rotational (torsional) support to the rail, so that the force at wheel–rail contact is distributed on a length around 5 ÷ 6 m, obtaining a stiffness in the lowest range. Another advantage of ERS is the reduction in the noise radiated by the rail, since the only exposed surface is the one related to railhead [29]. The main drawback of an ERS is the cost, mainly related to the elastomeric material, and the maintenance related to the rail’s substitution. For this reason, the ERS is used for lines with limited lengths, such as tramway lines, but application to rail systems also exist for bridges. A non-linear model for the frequency- and preload-dependent dynamic properties of the ERS is beneficial, for instance, for the installation case on a railway bridge, to predict the rail deflection, the transmitted force to the bridge, and the consequent noise emission with a single time domain train–track dynamic interaction simulation.

The present work is organised as follows. The first part of the paper illustrates the laboratory tests on a full-scale sample of the reference ERS and presents the results in terms of the equivalent stiffness and damping of the rail fastening system of elastomeric material as a function of frequency for different loading levels. In the second part, the parameters of a modified Zener model are identified and validated against the experimental results for the frequency- and preload-dependent stiffness and damping of the rail fastening system. The main focus is on the capability to represent damping in the analysed frequency range. Based on the validated model, a model of unit length of the ERS is developed. Finally, some numerical experiments are carried out with time domain simulation to illustrate the effect of the modelled non-linear dynamic behaviour of the elastomeric element on both the transient and steady-state response of the track.

## 2. Laboratory Tests

**Figure 1:**
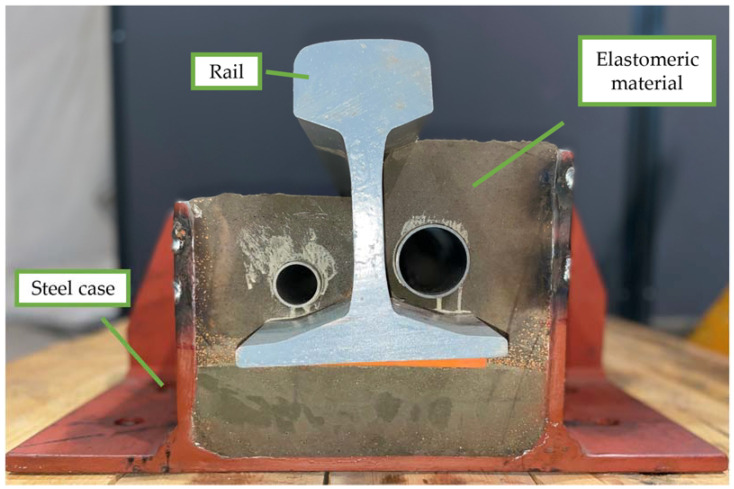
Section view of the reference Embedded Rail System (ERS).

The laboratory tests were performed on a full-scale 750 mm long sample of an ERS in the labs of the Department of Mechanical Engineering of Politecnico di Milano. The section view of the ERS is presented Figure 1. The ERS rail fastening system possesses a nominal static vertical stiffness of 50 MN m^−2^ (for unit length of the ERS), in which the rail (UIC 60) is enclosed in a poured-in-place polymer-based compound with cork filling material in a metallic case. The elastomeric material distribution is asymmetric and two plastic tubes with different diameters are inserted. The way in which the volume of elastomeric material contributes to the stiffness in vertical, lateral, and torsional stiffness changes accordingly. For instance, looking at the elastomeric volume, it can be noticed that the vertical stiffness is mainly due to the compression of the volume under the rail’s foot, in conjunction with the shear deformation of the two volumes at each side of rail web. It is reasonable to expect that the continuous rail fastener, made of the elastomeric material, has stiffness and damping dependent on static preload and frequency.

**Figure 2:**
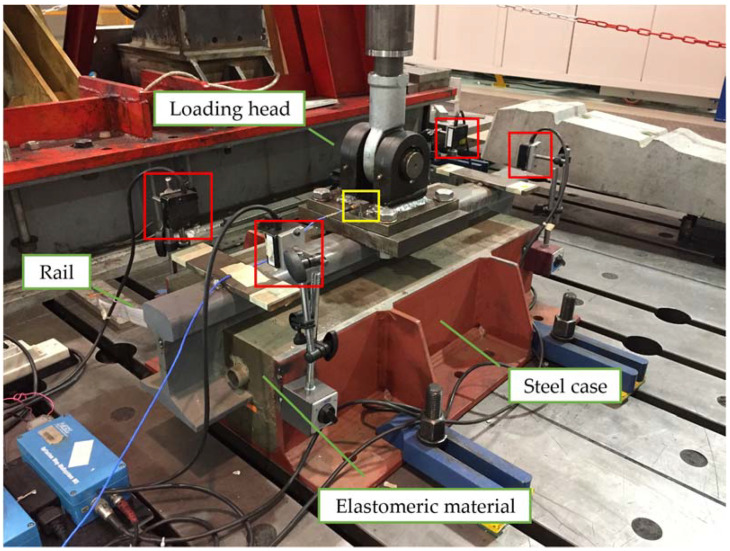
Test rig for full-scale experiments on a 750 mm long ERS sample to determine the dynamic behaviour in vertical direction. Red boxes: laser displacement transducers for vertical motion measurement. Yellow box: accelerometer for loading head acceleration measurement.

The test rig to determine the dynamic properties of the elastomeric materials in vertical direction is presented in Figure 2.

The tested configuration was proposed for the deck of a steel box girder bridge, in which the steel case of the ERS is directly connected to the upper plate of the deck. For the purpose of the test, the steel case was fixed to the ground. The rail was forced vertically by a hydraulic actuator controlled in force, while the displacement was measured by means of four non-contact laser displacement transducers (MEL Mikroelektronik GmbH, Eching, Germany), located at the rail’s extremities. The mean value of the four displacement signals was used as reference displacement at the point of application of the force. The latter was measured by a load cell located between actuator’s piston and loading head. Compensation of inertia terms due to the mass of the loading head was applied by means of acceleration measurement, also shown in the picture.

Several steps of the preload were considered. A first series of test was carried out, applying a mono-harmonic force, with a given static pre-load, at different frequencies. The test parameters are summarised in Table 2.

**Figure 3:**
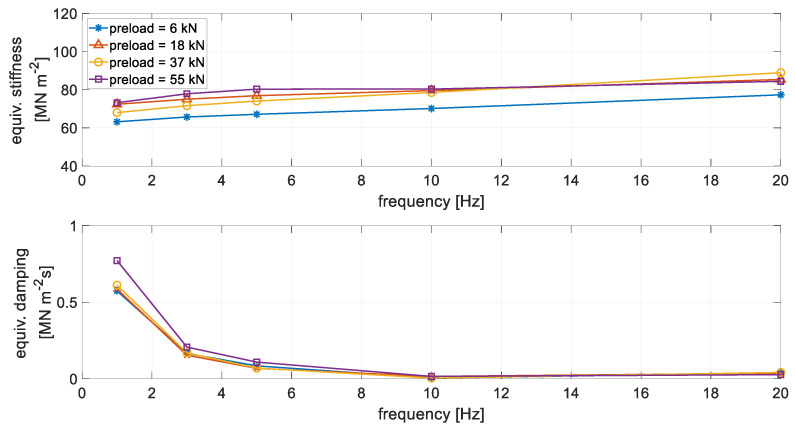
Equivalent stiffness (**upper** plot) and viscous damping (**lower** plot) of unit length of the elastomeric material of the ERS in function of frequency and preload.

The summary of the obtained results is reported in Figure 3, in terms of equivalent stiffness (upper plot) and equivalent viscous damping (lower plot), which are obtained considering the in-phase and quadrature components of the rail displacement, respectively, with respect to the mono-harmonic actuator force [24].

It can be observed that, as usual, the equivalent stiffness showed some increase with the frequency. Moreover, there was some degree of dependence on the preload of the applied force, especially in the lowest values of the investigated frequency range. On the other hand, the equivalent viscous damping coefficient strongly decreased as frequency increased, showing a much lower dependence on the preload.

The static stiffness of unit length of the ERS was also tested by applying a slowly increasing quasi-static load by the hydraulic actuator, and a value of 60 MN m^−2^ was measured.

The test rig was similar to the one explained in the direct method reported in the standard for the determination of stiffness of rail fastening systems [30]. With the current test rig set-up, the dynamic behaviour of the elastomeric element was computed by the rail head displacement and the applied force, considering the compensation of the rail inertia, considered as a rigid mass in the examined frequency range. Consequently, the characterised dynamic properties can be attributed to the unit length of the elastomeric material.

## 3. Numerical Models

Once the dynamic properties of the elastomeric material of the ERS were investigated experimentally, a modified Zener model for its frequency- and preload-dependent stiffness and damping was identified and validated with the laboratory test results. Similar to the determination of the dynamic properties, the model was also developed for unit length of the elastomeric element. The model of unit length of the ERS was subsequently set up with which time domain simulations were performed to illustrate the effect of the non-linear dynamic behaviour of the elastomeric elements in the train–track dynamic interaction.

### 3.1. Non-Linear Mechanical Model of Elastomeric Material

**Figure 4:**
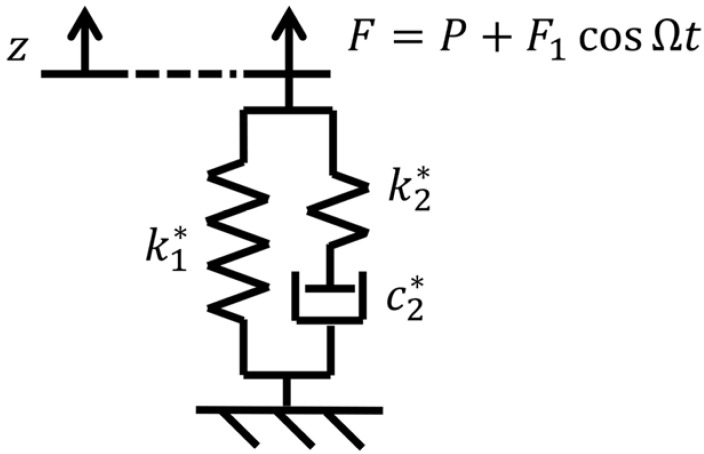
Non-linear macro-mechanical model of unit length of elastomeric material with preload-dependent parameters.

The macro-mechanical model of unit length of the elastomeric material of the ERS was a modified Zener model [25,26] and its structure is presented in Figure 4.

The variable *z* represents the deformation of the elastomeric material, and the *F* represents the external force to realise the deformation. In the case of the described laboratory tests, the applied external force had two components: the static preload *P* and the mono-harmonic force *F*_1_ cosΩ*t*. The reaction force generated by the elastomeric material had the same magnitude and an opposite direction of the external force. Parameters *k*_1_*, *k*_2_* and *c*_2_* stand for the values of the springs and damper, respectively. The original Zener model was linear in its formulation; the non-linearity is herein included considering the effect of the static preload as a simple multiplication factor of the base parameter (stiffness or damping), which are described as:

(1) $$\begin{array}{c} k_{1}^{*}=k_{1,0}{\left(1+P/P_{0}\right)}^{\chi },\\ k_{2}^{*}=k_{2,0}{\left(1+P/P_{0}\right)}^{\chi },\\ c_{2}^{*}=c_{2,0}{\left(1+P/P_{0}\right)}^{\chi }. \end{array}$$

where *k*_1,0_, *k*_2,0_, and *c*_2,0_, are constant reference values of *k*_1_*, *k*_2_*, and *c*_2_*; *P* is the static preload applied on the ERS; *F*_1_ is the amplitude of the dynamic load; Ω is the dynamic load frequency with the unit of rad/s; *P*_0_ is a constant reference preload; and *x* is a non-dimensional constant number. All model parameter values refer to unit length of the elastomeric material of the ERS.

The equivalent stiffness (*k_equiv._*) and damping (*c_equiv._*) of the non-linear macro-mechanical model, for a certain static preload and dynamic force frequency, are computed as:

(2) $$\begin{array}{l} k_{equiv.}\left(P,\Omega \right)=k_{1}^{*}+k_{2}^{*}\frac{{\left(c_{2}^{*}\Omega /k_{2}^{*}\right)}^{2}}{1+{\left(c_{2}^{*}\Omega /k_{2}^{*}\right)}^{2}},\\ c_{equiv.}\left(P,\Omega \right)=c_{2}^{*}\frac{1}{1+{\left(c_{2}^{*}\Omega /k_{2}^{*}\right)}^{2}}. \end{array}$$

The values of the model parameters, *k*_1,0_, *k*_2,0_, *c*_2,0_, and *x*, are determined through a minimisation procedure of the difference between the modelled and experimental equivalent stiffness and damping for all test frequencies and preloads. The constant reference preload *P*_0_ is set as 64 kN. The objective function of the minimisation procedure is defined as:

(3) $$\begin{array}{ll} err=\sum_{i=1}^{n_{P}}\sum_{j=1}^{n_{\Omega }} & [{\left(k_{equiv.,num.}\left(P_{i},\Omega_{j}\right)-k_{equiv.,exp.}\left(P_{i},\Omega_{j}\right)\right)}^{2}\\ & +10{\left(c_{equiv.,num.}\left(P_{i},\Omega_{j}\right)-c_{equiv.,exp.}\left(P_{i},\Omega_{j}\right)\right)}^{2}], \end{array}$$

where *i* and *j* are the indices of the preload case and test frequency, respectively; *n_p_* and *n*_Ω_ are the total number of the preload cases and test frequencies, respectively; *k_equiv.,num._* and *c_equiv.,num._* are the equivalent stiffness and damping computed by the numerical model; and *k_equiv.,exp._* and *c_equiv.,exp._* are the equivalent stiffness and damping computed by the experimental results. A weight of 10 was applied to the modelling error of the equivalent damping with respect to the equivalent stiffness for a better performance of the minimisation procedure due to the difference between the orders of magnitude of the two parameters. The identified model parameter values are presented in Table 3.

**Figure 5:**
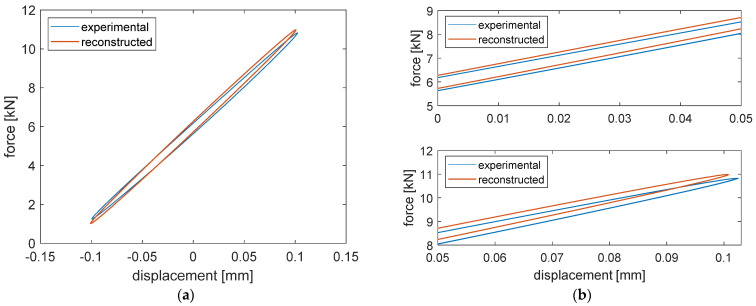

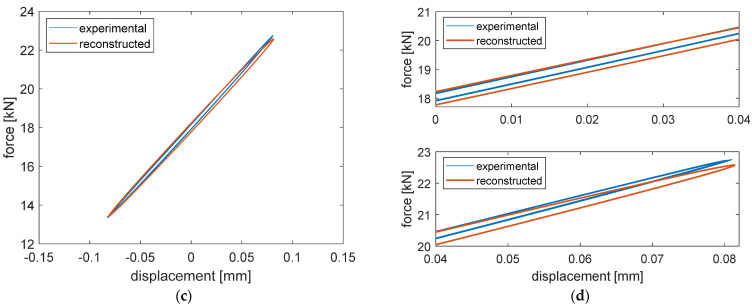
Example of force–displacement cycles with applied mono-harmonic force: (**a**) mono-harmonic force at 1 Hz with preload of 6 kN, full cycle; (**b**) mono-harmonic force at 1 Hz with preload of 6 kN, detailed presentations in the displacement range of 0 ÷ 0.05 mm (upper plot) and 0.05 ÷ 0.1 mm (lower plot); (**c**) mono-harmonic force at 5 Hz with preload of 18 kN, full cycle; (**d**) mono-harmonic force at 5 Hz with preload of 18 kN, detailed presentations in the displacement range of 0 ÷ 0.04 mm (upper plot) and 0.04 ÷ 0.08 mm (lower plot). Both tests are performed with a dynamical amplitude of 5 kN.

The force–displacement cycles were reconstructed numerically using the identified model parameters and compared to the experimental ones in Figure 5 for two test conditions. Note that when the numerical results are compared to the experimental ones, the model parameter values, used to obtain the numerical results, refer to a 750 mm segment of the ERS.

**Figure 6:**
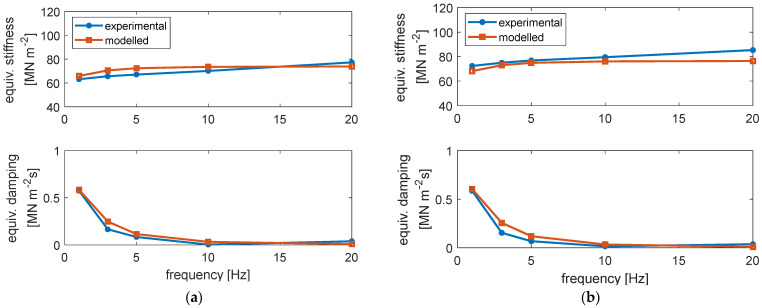

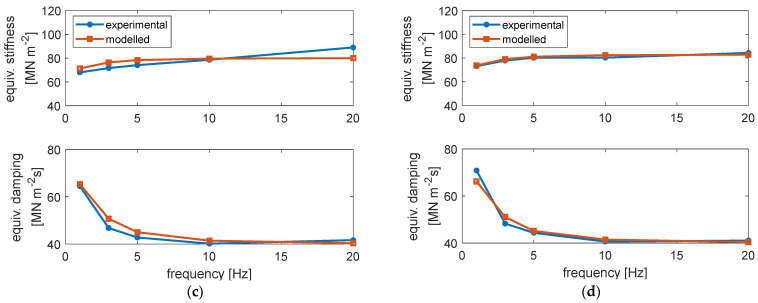
Modelled and experimental equivalent stiffness (upper plot of each part) and viscous damping (lower plot of each part) of unit length of the elastomeric element of the ERS: (**a**) preload of 6 kN; (**b**) preload of 18 kN; (**c**) preload of 37 kN; (**d**) preload of 55 kN.

The similarity of the measured and reconstructed loops indicates the ability of the model to reproduce the equivalent stiffness and damping at different frequencies and preloads. For a better illustration of the entire ensemble of test results, in Figure 6, the experimental and modelled equivalent stiffness (upper plot of each part) and viscous damping (lower plot of each part) of unit length of the elastomeric element of all test conditions are compared.

It can be observed that the modelled equivalent stiffness and viscous damping are quite similar to the experimental ones, in terms of both the absolute values and the variation trend as in function of preload and frequency. The best comparison is found for the damping, while for the stiffness, the maximum preload is better matched with respect to the lower preloads. It is worth noting that the modelled equivalent stiffness and damping are derived of different test conditions but are computed by a single set of model parameters. Therefore, it is acceptable that not all modelled data match the experimental ones.

**Figure 7:**
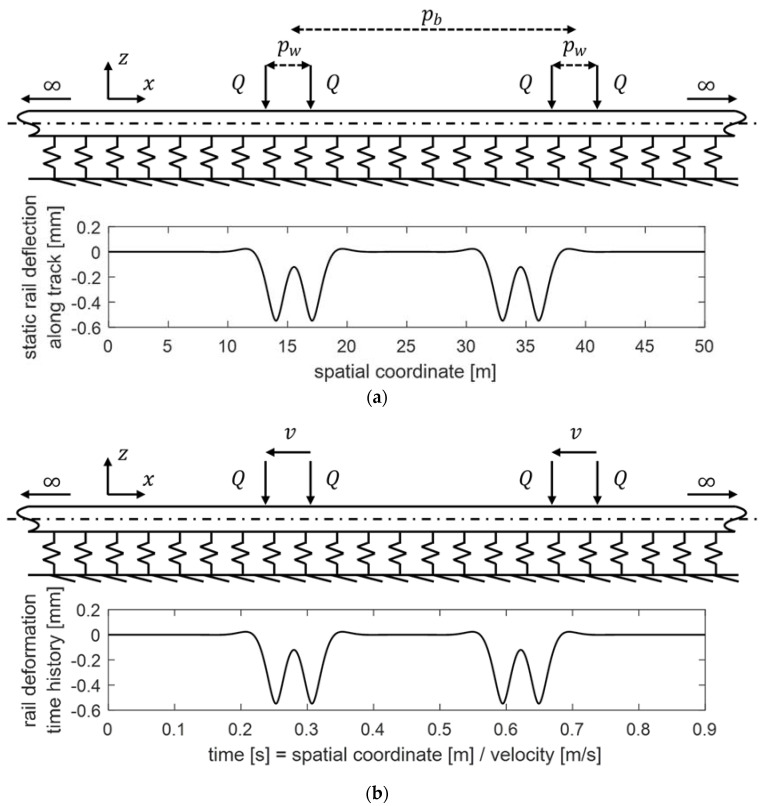
(**a**) Static rail deflection distribution along the track obtained with a Winkler model with the wheelset loads. (**b**) Elastomeric material deformation time history converted from the static rail deformation distribution along the track with a constant train velocity (*v* = 200 km/h).

In order to verify whether the model could predict the dynamic behaviour of the elastomeric element, the reaction force generated by the elastomeric element of the 750 mm ERS sample to a specific rail vertical displacement time history is predicted by performing a time domain simulation with the developed model and compared to the corresponding experimental result. Referring to the scheme of the model presented in Figure 4, the input of the simulation is the deformation time history of the elastomeric material *z*, while the output is the external force *F* to realise such deformation (the reaction force has the same magnitude and an opposite direction of the external force). The rail displacement time history is obtained by converting the static rail deflection distribution along the track, given by a single-beam Winkler model, on which wheelset loads are modelled as a sequence of discrete loads, with a constant train velocity to simulate the passage of a train. The scheme of the Winkler model with the wheelset loads is shown in Figure 7a together with the resultant static rail deflection distribution along the track, whose conversion to the deformation time history of the elastomeric material is illustrated in Figure 7b. The values of the wheelset loads *Q* (110 kN), wheelbase *p_w_* (3 m), and bogie base *p_b_* (19 m) refer to a passenger coach of ETR 500. The train velocity *v* is set at 200 km/h.

The profile of the rail deformation time history is equivalent to that of the static rail deflection distribution along the track—the only difference is the conversion from the spatial coordinate to time through the constant velocity.

The external force can be numerically integrated with the following differential equation, which can be obtained from Figure 4, that describes the relationship between the external force *F* and the deformation of the elastomeric material *z:*

(4) $$\dot{F}\left(t\right)=\left(k_{1}^{*}+k_{2}^{*}\right)\dot{x}\left(t\right)+\frac{k_{1}^{*}k_{2}^{*}}{c_{2}^{*}}x\left(t\right)-\frac{k_{2}^{*}}{c_{2}^{*}}F\left(t\right).$$

The calculations of *k*_1_*, *k*_2_*, and *c*_2_* are presented in Equation (1). Since the external force *F*(*t*) to be integrated is not a combination of a static preload and a mono-harmonic force, the static preload *P* in the calculation of *k*_1_*, *k*_2_*, and *c*_2_* are substituted by the instantaneous value of the external force *F*(*t*).

A corresponding laboratory test with the same input rail vertical displacement time history was performed with the test rig shown in Figure 2, and the external force to realise the displacement was measured.

**Figure 8:**
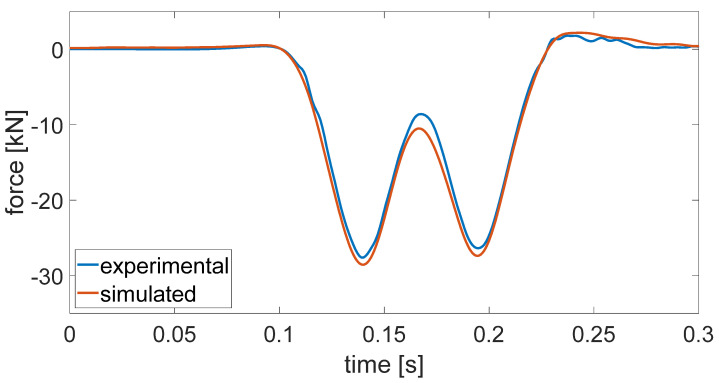
Comparison of the experimental and numerically simulated external force time histories applied on the 750 mm long ERS sample to realise a rail displacement simulating a train passage (ETR 500 coach at 200 km/h).

The experimental and numerically simulated external force time histories are compared in Figure 8. The visualisation of the time history was limited to a time interval corresponding to the passage of a single bogie because it was verified that the portion of the time history related to the passage of a single bogie is independent from the other ones.

The simulated external force was highly similar to the experimental one. Regarding the trend of the time history, it correctly simulated that the second valley of the external force had a lower magnitude than the first one and the positive force after the two valleys corresponding to the passages of the two wheelsets. Furthermore, the simulated external force was very close to the experimental one in terms of the absolute value.

### 3.2. Models of Unit-Length of Reference ERS

**Figure 9:**
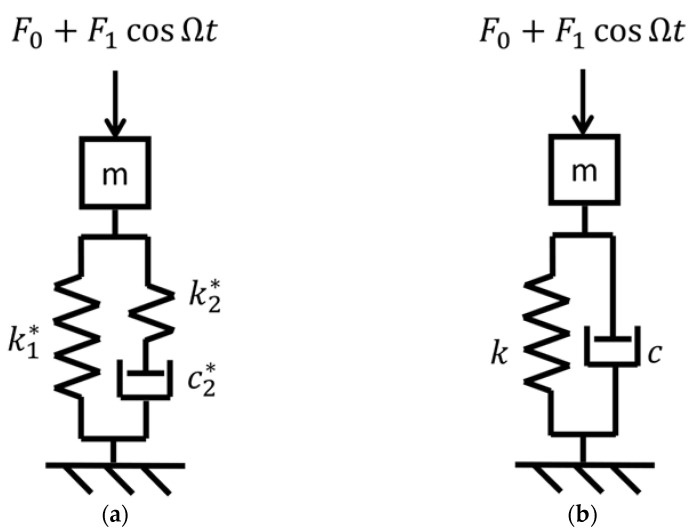
Mechanical model of unit length of the reference ERS subjected to external force for time domain simulation: (**a**) non-linear Zener model with preload-dependent parameters; (**b**). linear Kelvin-Voigt model.

After developing the model of the elastomeric element, the model of unit length of the ERS was developed, where a mass representing the rail was added on the top of the model of the elastomeric element. In order to investigate the effect of the preload- and frequency-dependent stiffness and viscous damping of the elastomeric element on train–track dynamic interaction, its effect on the dynamic behaviour of the reference ERS was firstly studied. Specifically, the response of the ERS to a harmonic force applied on the rail head was simulated (the scheme of the simulation is illustrated in Figure 9a). For comparison, simulations were also performed with a linear spring–damper model (Figure 9b), where the dependence of dynamic properties on preload and frequency were not considered, as it usually happens for classical track model. The stiffness and damping of the linear spring–damper model were 80 MN m^−2^ and 10^−3^ MN m^−2^ s, respectively, referring to unit length of the ERS. The chosen stiffness value is regarded as representative of different frequencies and preloads according to the experimental data (see Figure 3).

**Figure 10:**
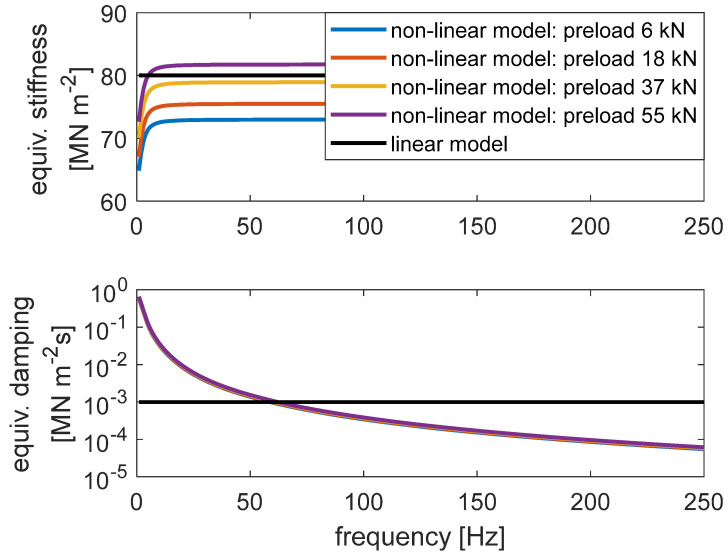
Equivalent stiffness (**upper** plot) and viscous damping (**lower** plot) computed by the non-linear and linear mechanical model of unit length of the elastomeric element of the reference ERS.

According to the composition of the two models of unit-length of the reference ERS, the dynamic behaviour of the models was apparently strongly influenced by the assigned values of the model parameters. Therefore, for an easier interpretation of the simulation results, in Figure 10, the equivalent stiffness (upper plot) and viscous damping (lower plot) computed by the non-linear and linear models of unit length of the elastomeric element of ERS in the frequency range of 0 ÷ 250 Hz are compared. It can be observed that the dynamic stiffness of the non-linear model, with a constant preload, increased rapidly at a low-frequency range, i.e., approximately 0 ÷ 20 Hz, and then approached an asymptote. This asymptote increased with the preload according to the model’s formulation. On the contrary, the linear model had a fixed stiffness regardless of the frequency and preload. Concerning the equivalent damping, that of the non-linear model decreased from 1 MN m^−2^ s to 10^−4^ MN m^−2^ s (note that the plot is in logarithmic scale) in the considered frequency range while the linear model had a constant value of 10^−3^ MN m^−2^ s. From this last figure, it is clear that the use of a viscous damping not depending on frequency can be set only corresponding to one frequency, and as a consequence, there is an overestimation of the damping beyond the setting frequency and an underestimation below the same value.

**Figure 11:**
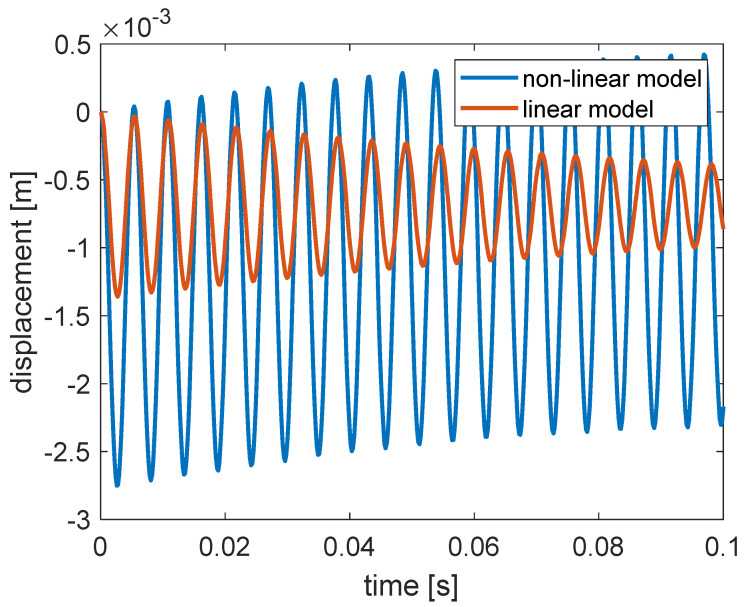
Free responses simulated by the non-linear and linear mechanical model of unit length of the reference ERS subjected to a constant force of 55 kN with null initial conditions.

The large difference in the viscous damping of the two models can have a strong effect on the simulated rail response, both on the transient phase and the steady state phase. Figure 11 represents the simulated free response of the rail subjected to a constant force of 55 kN with null initial conditions.

The response of the linear model had a period of approximately 0.0054 s (185 Hz) and decreased logarithmically with time. The 185 Hz corresponds to the natural frequency of the linear model of the ERS if it was regarded as a single degree of freedom system. Instead, the response of the non-linear model had a shorter period and a much larger amplitude, even though the simulation conditions are identical. Furthermore, the response seemed to not decrease with time in the presented time window. In fact, the response obtained with the non-linear model also decayed with time, but the decay rate was much lower than that of the linear model. It took 7.6 s for the non-linear model to reach a 90% decrease in the vibration amplitude while the time needed for the linear model was about 0.3 s. This can be attributed to the much lower equivalent viscous damping value of the non-linear model compared to the linear one in the frequency range around 185 Hz, as shown in the lower plot of Figure 10.

**Figure 12:**
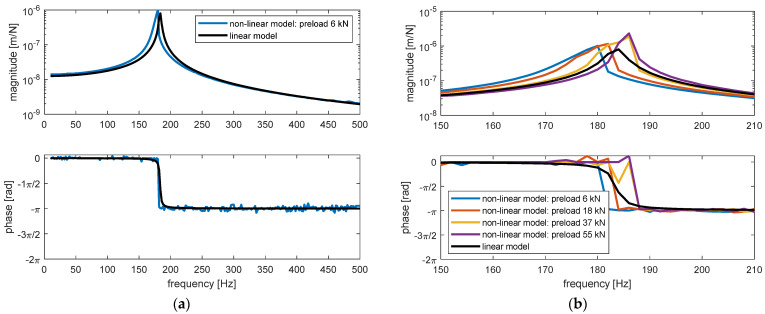
Amplitude ratio (upper plot of each part) and phase delay (lower plot of each part) of the steady-state responses of the rail and the external harmonic force, obtained with time domain simulation by linear and non-linear mechanical models of unit length of the ERS: (**a**) preload of 6 kN and dynamic force amplitude of 1 kN; (**b**) preload of 6 kN, 18 kN, 37 kN, 55 kN, and dynamic force amplitude of 1 kN.

In Figure 12, results of the steady state response of the rail to the harmonic force (see Figure 9) computed by the non-linear and linear models are presented.

For the non-linear model, time domain simulation was performed where the rail was subjected to a harmonic force with preload, as shown in Figure 9a. The ratio of the amplitude between the steady-state response, synchronous with the forcing frequency, and that of the external force was calculated (upper plot of each part) as well as the phase delay between the two signals (lower plot of each part). Both elaborated results were compared to those of the linear model. In Figure 12a, the results of the non-linear model are obtained with a preload of 6 kN and an amplitude of the harmonic force equal of 1 kN. The external harmonic frequency varies from 10 Hz to 500 Hz with a step of 2 Hz. The result of the linear model is substantially its frequency response function in terms of the rail displacement and is obtained with frequency domain calculation. Regarding the magnitude, the results can be roughly divided into three sections according to the frequency response function of the linear model: a quasi-static zone (approximately 0 ÷ 150 Hz), a resonance zone (approximately 150 ÷ 200 Hz), and a seismic zone (over 200 Hz). The curve of the non-linear model is different from the linear one, mainly in the quasi-static zone and the resonance zone. More specifically, the difference in the resonance zone is more obvious. According to the interpretation of the frequency response function of the linear model, the quasi-static zone is dominated by the stiffness of the model, the resonance zone is dominated by the viscous damping, and the seismic zone is dominated by the mass property. Consequently, it is reasonable that the two curves coincide in the seismic zone since the mass per unit length of the two models are identical. Similarly, the difference is mainly in the resonance zone since the most principal difference of the dynamic behaviour of the two models is the viscous damping (according to the lower plot of Figure 10). Meanwhile, the difference regarding the frequency of the peak values, about 4 Hz, is limited, since the stiffness values, unlike the damping values, of the two models have the same order of magnitude. Regarding the phase delay, the curves obtained from the two models are similar. That of the linear model decreased from 0 rad to −π, crossing the resonance zone near 185 Hz.

In Figure 12b, the results of the non-linear model obtained with different preloads are presented (6 kN, 18 kN, 37 kN, 55 kN). The magnitude of the harmonic force equals 1 kN for all preloads. The external harmonic frequency varies from 150 Hz to 210 Hz with a step of 2 Hz. The result of the linear model is identical to the one in Figure 12a. The frequency range is the resonance zone according to the linear model. Regarding the magnitude ratio, the higher the preload, the higher the peak value and higher the corresponding frequency. The peak values of the curves obtained with the non-linear model are approximately 3 times that of the linear model (note that the plot is in logarithmic scale).

**Figure 13:**
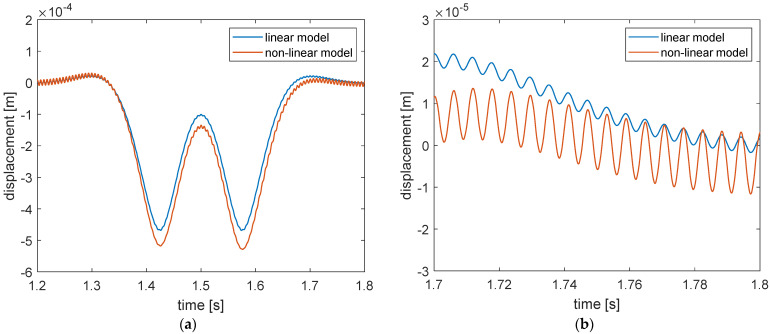
Rail responses predicted by the non-linear and linear models of unit length of the reference ERS subjected to a load simulating a train passage (ETR 500 coach at 72 km/h) and a dynamic force caused by a rail roughness with a wavelength of 120 mm (170 Hz) with an amplitude of 20 N: (**a**) time window of 1.2 ÷ 1.8 s (centred at the instant of the passage of a bogie); (**b**) time window of 1.7 ÷ 1.8 s (for a better visualisation of the high-frequency vibration).

To study the effect of the dynamical properties of the elastomeric element of the ERS on the rail response in the case of the train–track dynamic interaction, a time domain simulation similar to the one presented in Figure 9 was performed with both the linear and non-linear model. The model compositions remained invariant while the constant force component was substituted by a quasi-static force time history simulating the passage of a train (ETR 500 coach at 72 km/h). The dynamic force component had an amplitude of 20 N and a frequency of 170 Hz, simulating a dynamic force caused by a rail roughness with a wavelength of 120 mm, which is a typical wavelength of short pitch corrugation. The results are presented in Figure 13 and limited to a 0.6 s time window centred at the time instant of the passage of a bogie in part (a). For a better visualisation of the high-frequency vibration, the time histories are limited to the time window of 1.7 ÷ 1.8 s in part (b).

Regarding the low-frequency response associated with the passage of the wheelsets (approximately 5 Hz), the non-linear model predicted a larger displacement due to the lower stiffness in the concerned frequency range. The difference between the maximum displacements was about 11%. For the high-frequency vibration associated with the dynamic force component, the amplitude obtained with the non-linear model was about 3 times that obtained with the linear model, which is quite close to the magnitude ratio presented in Figure 12. The effect is more observable before and after the passage of the wheelsets and less obvious during the passage due to the rapid large-scale displacement of the rail.

**Figure 14:**
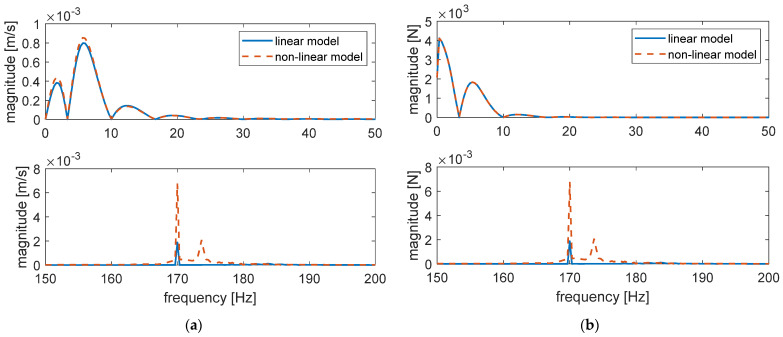
Spectra of the simulated vertical velocity of the rail obtained by the non-linear and linear models of unit length of the reference ERS subjected to a load simulating a train passage (ETR 500 coach at 72 km/h) and a dynamic force caused by a rail roughness with a wavelength of 120 mm (170 Hz) with an amplitude of 20 N: (**a**) spectra of the rail velocity; (**b**) spectra of the transmitted force. All spectra are calculated with a time window of 3 s.

To extend the effect of the modelled non-linear dynamic behaviour on the rail response to the study of railway issues, taking the rail noise emission and the transmitted force to the subgrade as example, the spectra of the rail velocity and the transmitted force are computed and presented in Figure 14a,b, respectively. The upper plots illustrate the spectra in the frequency range of 0 to 50 Hz while the lower plots illustrate the spectra in the frequency range of 150 to 200 Hz, where the high-frequency dynamical force is superposed. All spectra are based on a time window of 3 s centred at the instant of the passage of the bogie.

The low-frequency range contribution relates to the passage of the wheelsets, while the main peaks located at 170 Hz in the high-frequency range are associated with the superposed dynamical force representing the effect of the short pitch corrugation. Only for the spectra obtained with the non-linear model can a peak located around 173 Hz be observed, corresponding to the transient response of the rail.

The patterns of Figure 14a,b are almost identical with the two models in the low-frequency range, since the response of the rail is dominated by the equivalent stiffness, which is only slightly different. Meanwhile, the magnitude of the peaks at high frequency differs, since the equivalent viscous damping of the two models are remarkably different in the concerned frequency range.

## 4. Conclusions

In this paper, a macro-mechanical model of the elastomeric element of an Embedded Rail System (ERS) in the form of a modified Zener model was set up for reproducing the dynamic behaviour of the elastomeric element, that are characterised by laboratory tests. The test results demonstrated the dependence of the dynamic behaviour of the elastomeric element on frequency and preload. The macro-mechanical model, whose parameters are tuned by the test results, is able to represent the dynamic behaviour of the elastomeric element at different frequencies and preloads, with a unique model working in the time domain.

Then, a model of unit length of the ERS was developed based on the model of the elastomeric element. Time domain simulations were performed as numerical experiments to predict the response of the ERS to a harmonic force, together with a static preload, applied on the rail head, and the results were compared to those computed by a fully linear model. The results suggest that the dynamic properties of elastomeric elements, especially the viscous damping, have a strong influence on the response of the ERS. On the one hand, the constant stiffness value of the linear model has the same order of magnitude compared to those of the non-linear model at different frequencies and preloads in consideration. On the other hand, the constant damping value of the linear model is not comparable to that of the non-linear model in all the frequency ranges. Consequently, while the prediction of the frequency at which the rail response has the maximum value is limited (about 4 Hz), the prediction of the magnitude is rather different—about three times higher for the non-linear model—in terms of the vibration of the rail.

Lastly, in the time domain simulation of a train passage, the non-linear model predicted a higher low-frequency rail displacement associated with the passage of the wheelsets (about 11%) and a higher amplitude of the high-frequency vibration associated with the dynamic force component (about 2.5 times).

The simulation results obtained suggest that the nonlinear track behaviour needs to be carefully modelled when accurate prediction of the track vibration is required. To this end, the macro-mechanical models of track elastomeric elements can be developed from lab tests (considering different combinations of the test parameters, so as to cover typical operating conditions) and then included in train–track dynamic interaction models.

It is worth pointing out that it is important to choose an appropriate dimension of the test sample of the elastomeric elements for a correct characterisation of its dynamic properties. When it comes to the discrete track components such as the rail pad or the under sleeper pad, there is no need for such consideration. However, it is necessary for the continuous elastomeric element, such as the rail fastening system of the reference ERS. On the one hand, the test sample should be long enough to avoid the influence of the incompressibility of the elastomeric material on the dynamic property characterisation. On the other hand, it should be short enough so that the loading component, the rail in the current study, always moves as a rigid body and the characterised dynamic property can be attributed to the unit length of the elastomeric material.

Some aspects of the current study can still be improved or further investigated in the future. The highest excitation frequency of the laboratory tests was 20 Hz due to the limitation of the hydraulic actuator. An extension of the characterisation frequency range versus the high-frequency direction, such as through impact excitations, would extend the validation frequency range of the numerical model and thus increase its reliability in the high-frequency range. For some specific application cases of the ERS, such as in the regions where the environment temperature experiences a large variation during the operation time, it is worth characterising the dynamic properties in function of the environment temperature.

## Figures and Tables

**Table 1:** Concerned frequency ranges of the various issues related to the dynamic behaviour of the railway track.

Railway Issues	Concerned Frequency Range (Hz)
Rail deflection	5 ÷ 20
Transmitted forces	10 ÷ 250
Railway rolling noise	20 ÷ 10,000

**Table 2:** Parameters of the tests to determine the dynamic properties of the elastomeric materials in the vertical direction.

Test Parameter	Unit	Value
Static preload	kN	6, 18, 37, 55
Mono-harmonic force frequency	Hz	1, 3, 5, 10, 20
Mono-harmonic force amplitude	kN	5

**Table 3:** Identified parameter values of the non-linear mechanical model of unit length of elastomeric material.

Parameter	Unit	Value
*k*_1,0_	MN m^−2^	62
*k*_2,0_	MN m^−2^	9.6
*c*_2,0_	MN m^−2^ s	0.7
*x*	/	0.21
*P*_0_	kN	64

## Data Availability

The data presented in this study are available on request from the corresponding author. The data are not publicly available due to confidential reasons.
